# 6,8-Dibromo-5-hydr­oxy-4-oxo-2-phenyl-4*H*-chromen-7-yl acetate

**DOI:** 10.1107/S1600536809005431

**Published:** 2009-02-21

**Authors:** Angannan Nallasivam, Munirathinam Nethaji, Nagarajan Vembu, Venkatraman Ragunathan, Nagarajan Sulochana

**Affiliations:** a Department of Chemistry, National Institute of Technology, Tiruchirappalli 620 015, India; b Department of Inorganic and Physical Chemistry, Indian Institute of Science, Bangalore 560 012, India; c Department of Chemistry, Urumu Dhanalakshmi College, Tiruchirappalli 620 019, India; d Department of Chemistry, Kandasamy Kandar College, Velur 638 182, India

## Abstract

In the title compound, C_17_H_10_Br_2_O_5_, the chromene ring is almost planar with minimal puckering [total puckering amplitude = 0.067 (4) Å]. The dihedral angle between chromeme ring system and phenyl ring is 3.7 (2)°. The crystal structure is stabilized by intermolecular C—H⋯O inter­actions and an intramolecular O—H⋯O hydrogen bond also occurs.

## Related literature

For the biological and pharmacological properties of benzopyrans and their derivatives, see: Brooks (1998 ▶); Hatakeyama *et al.* (1988 ▶)); Hyana & Saimoto (1987 ▶); Tang *et al.* (2007 ▶). For the importance of 4*H*-chromenes, see: Liu *et al.* (2007 ▶); Wang, Fang *et al.* (2003 ▶); Wang, Zhang *et al.* (2003 ▶). For hydrogen bonding, see: Bernstein *et al.* (1995 ▶); Desiraju (1989 ▶); Desiraju & Steiner (1999 ▶); Etter (1990 ▶).
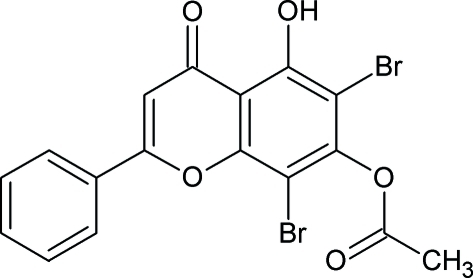


## Experimental

### 

#### Crystal data

C_17_H_10_Br_2_O_5_
*M* *_r_* = 454.07
Monoclinic, 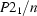

*a* = 14.072 (3) Å
*b* = 5.5586 (13) Å
*c* = 21.333 (5) Å
β = 104.501 (4)°
*V* = 1615.6 (7) Å^3^
*Z* = 4
Mo *K*α radiation
μ = 5.04 mm^−1^
*T* = 293 K
0.55 × 0.23 × 0.12 mm

#### Data collection

Bruker SMART APEX CCD diffractometer
Absorption correction: multi-scan (*SADABS*; Sheldrick, 1998 ▶) *T*_min_ = 0.157, *T*_max_ = 0.547
13326 measured reflections
3773 independent reflections
2245 reflections with *I* > 2σ(*I*)
*R*_int_ = 0.046

#### Refinement

*R*[*F* ^2^ > 2σ(*F* ^2^)] = 0.044
*wR*(*F* ^2^) = 0.105
*S* = 0.99
3773 reflections
221 parameters
H-atom parameters constrained
Δρ_max_ = 0.57 e Å^−3^
Δρ_min_ = −0.37 e Å^−3^

### 

Data collection: *SMART* (Bruker, 2007 ▶); cell refinement: *SAINT* (Bruker, 2007 ▶); data reduction: *SAINT*; program(s) used to solve structure: *SHELXS97* (Sheldrick, 2008 ▶); program(s) used to refine structure: *SHELXL97* (Sheldrick, 2008 ▶); molecular graphics: *PLATON* (Spek, 2009 ▶); software used to prepare material for publication: *SHELXL97*.

## Supplementary Material

Crystal structure: contains datablocks I, global. DOI: 10.1107/S1600536809005431/er2060sup1.cif

Structure factors: contains datablocks I. DOI: 10.1107/S1600536809005431/er2060Isup2.hkl

Additional supplementary materials:  crystallographic information; 3D view; checkCIF report

## Figures and Tables

**Table 1:** Hydrogen-bond geometry (Å, °)

*D*—H⋯*A*	*D*—H	H⋯*A*	*D*⋯*A*	*D*—H⋯*A*
O12—H12⋯O11	0.82	1.86	2.584 (4)	147
C3—H3⋯O11^i^	0.93	2.57	3.497 (4)	171
C24—H24⋯O11^i^	0.93	2.48	3.387 (5)	166
C17—H17*A*⋯O16^ii^	0.96	2.58	3.309 (6)	133

Symmetry codes: (i) 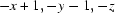; (ii) 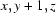.

**Fig. 1.**
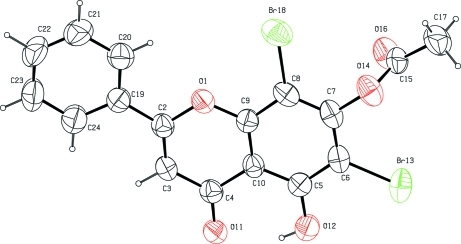
The asymmetric unit with the atoms labelled and displacement ellipsoids depicted at the 50% probability level for all non-H atoms. H-atoms are drawn as spheres of arbitrary radius.

**Fig. 2.**
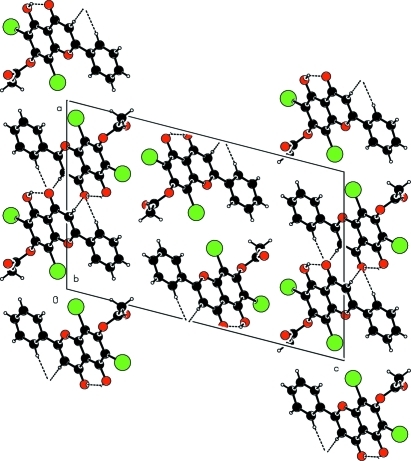
The molecular packing viewed down the b-axis. Dashed lines represent the weak C–H···O interactions within the lattice.

**Fig. 3.**
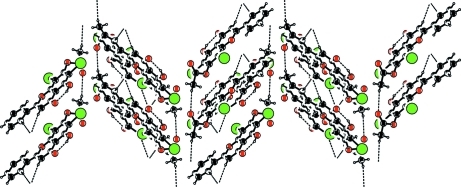
Cooperative H-bonded network of O–H···O interactions viewed down the a-axis

## supplementary crystallographic information

### Comment

Chromenes (benzopyrans) and their derivatives have numerous biological and pharmacological properties (Tang *et al.*, 2007) such as antisterility (Brooks, 1998) and anticancer activity (Hyana & Saimoto, 1987). In addition, polyfunctionalized chromene units are present in numerous natural products (Hatakeyama *et al.*, 1988). 4*H*-chromenes are important synthons for some natural products (Liu *et al.*, 2007). As a part of our structural investigations on 4*H*-chromene derivatives and compounds containing the benzopyran fragment, the single-crystal X-ray diffraction study on the title compound was carried out.

The chromene ring is almost planar similarly as those found in the related chromene derivatives (Wang, Fang *et al.*, 2003; Wang, Zhang *et al.*, 2003). The total puckering amplitude of the chromene ring is 0.067 (4) Å in the title structure. The interplanar angle between the chromene ring and the 2-phenyl ring is 3.7 (2)° thereby indicating the almost coplanar arrangement (Fig. 1). The OCOCH_3_ substituent at C7 is non-coplanar with the chromene ring as discerned from the interplanar angle of 87.4 (1)°.

The crystal structure is stabilized by the interplay of C–H···O and O–H···O interactions (Fig. 2 & Table 1). The H-bond distances agree with those reported in literature (Desiraju & Steiner, 1999; Desiraju, 1989). The C20–H20···O1 interaction generates a motif of graph set (Bernstein *et al.*, 1995; Etter, 1990) S(5). An S(6) motif is formed by O12–H12···O11 interaction. This interaction is also responsible for the formation of a cooperative H-bonded network (Fig. 3). The C3–H3···O11^i^ and C24–H24···O11^i^ interactions constitute a pair of bifurcated acceptor bonds generating a ring of graph set *R*^1^_2_(7). There are no significant C—H···π and π···π interactions.

### Experimental

In to the RBF, a suspension of chrysin (1 g, 3.93 mmol) and potassium carbonate (1.64 g, 11.81 mmol) in dimethyl formamide (10 ml) were added. The reaction mixture was heated to 383 K for 2–3 hrs. The reaction mixture was cooled to 313 K and acetyl chloride (1.23 g, 15.74 mmol) was slowly added with the help of dropping funnel. The reaction mixture was maintained for 8–9 hr at 313 K and monitored by HPLC. After completion of the reaction, the contents were quenched with water and stirred for 30–45 min at 303 K. The crude solid obtained was filtered and washed with plenty of water followed by methanol and dried under vacuum at 343 K. The acetylated compound was then taken in RBF and dissolved in dichloromethane (10 ml) and cooled to 273 K. Bromine (0.6 ml, 11.81 mmol) was added dropwise over a period of 15–20 min. The reaction mixture was maintained at 273 K for 5–6 hr. After completion of the reaction, the reaction mixture was quenched in ice water and extracted with dichloromethane (10 ml) and purified by column chromatography using ethyl acetate: n-hexane (20:80). The crude brominated product was then dissolved in dichloromethane (10 ml) and equal amount of n-Hexane (10 ml). The clear solution was kept for a week without stirring. Diffraction quality needle shaped crystals of average size 0.23 mm were obtained which were filtered and washed with n-hexane and dried under vacuum at 343 K. Yield: 80%

### Refinement

All the H-atoms were observed in the difference electron density map. However, they were situated into idealized positions with C–H = 0.93 and 0.96 Å for aryl and methylene H, respectively and O–H = 0.82Å for hydroxyl H-atoms and and constrained to ride on their parent atoms with *U*_iso_(H)=1.2Ueq(C) for C—H and *U*_iso_(H)=1.5Ueq(O) for O—H.

### Crystal data

**Table:**

C_17_H_10_Br_2_O_5_	*F*(000) = 888
*M**_r_* = 454.07	*D*_x_ = 1.867 Mg m^−^^3^
Monoclinic, *P*2_1_/*n*	Melting point = 433–435 K
Hall symbol: -P 2yn	Mo *K*α radiation, λ = 0.71073 Å
*a* = 14.072 (3) Å	Cell parameters from 475 reflections
*b* = 5.5586 (13) Å	θ = 2.0–27.0°
*c* = 21.333 (5) Å	µ = 5.04 mm^−^^1^
β = 104.501 (4)°	*T* = 293 K
*V* = 1615.6 (7) Å^3^	Rectangular, brown
*Z* = 4	0.55 × 0.23 × 0.12 mm

### Data collection

**Table:**

Bruker SMART APEX CCD diffractometer	3773 independent reflections
Radiation source: fine-focus sealed tube	2245 reflections with *I* > 2σ(*I*)
graphite	*R*_int_ = 0.046
Detector resolution: 8.3 pixels mm^-1^	θ_max_ = 28.0°, θ_min_ = 2.0°
ω scans	*h* = −18→18
Absorption correction: multi-scan (*SADABS*; Sheldrick, 1998)	*k* = −7→7
*T*_min_ = 0.157, *T*_max_ = 0.547	*l* = −25→28
13326 measured reflections	

### Refinement

**Table:**

Refinement on *F*^2^	Primary atom site location: structure-invariant direct methods
Least-squares matrix: full	Secondary atom site location: difference Fourier map
*R*[*F*^2^ > 2σ(*F*^2^)] = 0.044	Hydrogen site location: inferred from neighbouring sites
*wR*(*F*^2^) = 0.105	H-atom parameters constrained
*S* = 0.99	*w* = 1/[σ^2^(*F*_o_^2^) + (0.0505*P*)^2^] where *P* = (*F*_o_^2^ + 2*F*_c_^2^)/3
3773 reflections	(Δ/σ)_max_ = 0.001
221 parameters	Δρ_max_ = 0.57 e Å^−^^3^
0 restraints	Δρ_min_ = −0.37 e Å^−^^3^

### Special details

**Table:**

Geometry. All e.s.d.'s (except the e.s.d. in the dihedral angle between two l.s. planes) are estimated using the full covariance matrix. The cell e.s.d.'s are taken into account individually in the estimation of e.s.d.'s in distances, angles and torsion angles; correlations between e.s.d.'s in cell parameters are only used when they are defined by crystal symmetry. An approximate (isotropic) treatment of cell e.s.d.'s is used for estimating e.s.d.'s involving l.s. planes.
Refinement. Refinement of *F*^2^ against ALL reflections. The weighted *R*-factor *wR* and goodness of fit *S* are based on *F*^2^, conventional *R*-factors *R* are based on *F*, with *F* set to zero for negative *F*^2^. The threshold expression of *F*^2^ > σ(*F*^2^) is used only for calculating *R*-factors(gt) *etc*. and is not relevant to the choice of reflections for refinement. *R*-factors based on *F*^2^ are statistically about twice as large as those based on *F*, and *R*- factors based on ALL data will be even larger.

### Fractional atomic coordinates and isotropic or equivalent isotropic displacement parameters (Å^2^)

**Table:**

	*x*	*y*	*z*	*U*_iso_*/*U*_eq_	
O1	0.75605 (17)	−0.0593 (5)	−0.00552 (13)	0.0490 (7)	
C2	0.6921 (3)	−0.2392 (7)	−0.03148 (18)	0.0441 (9)	
C3	0.6143 (3)	−0.2872 (7)	−0.00800 (19)	0.0444 (9)	
H3	0.5734	−0.4146	−0.0256	0.069 (6)*	
C4	0.5916 (3)	−0.1506 (7)	0.04288 (19)	0.0429 (9)	
C5	0.6440 (3)	0.1983 (7)	0.11713 (18)	0.0424 (9)	
C6	0.7094 (3)	0.3853 (7)	0.13882 (18)	0.0455 (9)	
C7	0.7897 (3)	0.4160 (7)	0.11257 (19)	0.0484 (10)	
C8	0.8070 (3)	0.2636 (7)	0.0658 (2)	0.0474 (10)	
C9	0.7396 (3)	0.0821 (7)	0.04300 (18)	0.0416 (9)	
C10	0.6588 (3)	0.0450 (6)	0.06832 (18)	0.0419 (9)	
O11	0.51819 (18)	−0.1904 (5)	0.06422 (13)	0.0506 (7)	
O12	0.5671 (2)	0.1687 (5)	0.14257 (14)	0.0550 (7)	
H12	0.5375	0.0458	0.1279	0.101 (9)*	
Br13	0.68667 (3)	0.60136 (8)	0.20112 (2)	0.06240 (17)	
O14	0.8497 (2)	0.6151 (5)	0.13072 (15)	0.0600 (8)	
C15	0.9279 (3)	0.5925 (8)	0.1845 (2)	0.0523 (10)	
O16	0.9461 (2)	0.4080 (6)	0.21218 (15)	0.0660 (8)	
C17	0.9808 (3)	0.8239 (8)	0.1978 (3)	0.0747 (15)	
H17A	0.9374	0.9448	0.2069	0.101 (9)*	
H17B	1.0033	0.8711	0.1607	0.101 (9)*	
H17C	1.0361	0.8062	0.2345	0.101 (9)*	
Br18	0.91815 (3)	0.30002 (10)	0.03298 (2)	0.06851 (18)	
C19	0.7192 (3)	−0.3583 (7)	−0.08556 (19)	0.0471 (10)	
C20	0.8013 (3)	−0.2850 (9)	−0.1052 (2)	0.0607 (12)	
H20	0.8397	−0.1587	−0.0841	0.069 (6)*	
C21	0.8258 (3)	−0.4012 (10)	−0.1563 (2)	0.0741 (14)	
H21	0.8810	−0.3525	−0.1694	0.069 (6)*	
C22	0.7703 (4)	−0.5856 (10)	−0.1876 (2)	0.0751 (14)	
H22	0.7879	−0.6629	−0.2217	0.069 (6)*	
C23	0.6888 (4)	−0.6571 (9)	−0.1692 (2)	0.0781 (15)	
H23	0.6507	−0.7828	−0.1908	0.069 (6)*	
C24	0.6626 (3)	−0.5442 (8)	−0.1186 (2)	0.0650 (12)	
H24	0.6065	−0.5930	−0.1065	0.069 (6)*	

### Atomic displacement parameters (Å^2^)

**Table:**

	*U*^11^	*U*^22^	*U*^33^	*U*^12^	*U*^13^	*U*^23^
O1	0.0447 (14)	0.0548 (18)	0.0464 (16)	−0.0054 (12)	0.0096 (12)	−0.0050 (14)
C2	0.044 (2)	0.046 (2)	0.039 (2)	0.0008 (17)	0.0044 (18)	−0.0001 (19)
C3	0.044 (2)	0.042 (2)	0.043 (2)	−0.0052 (18)	0.0043 (18)	−0.0035 (19)
C4	0.042 (2)	0.046 (3)	0.038 (2)	0.0027 (17)	0.0031 (17)	0.0030 (18)
C5	0.045 (2)	0.043 (2)	0.037 (2)	0.0052 (18)	0.0067 (18)	0.0068 (19)
C6	0.058 (2)	0.034 (2)	0.038 (2)	0.0010 (18)	0.0007 (18)	0.0031 (18)
C7	0.057 (2)	0.035 (2)	0.045 (2)	−0.0042 (19)	−0.003 (2)	0.005 (2)
C8	0.043 (2)	0.046 (3)	0.049 (3)	−0.0046 (18)	0.0034 (18)	0.009 (2)
C9	0.044 (2)	0.041 (2)	0.036 (2)	0.0044 (17)	0.0052 (17)	0.0021 (19)
C10	0.044 (2)	0.039 (2)	0.039 (2)	0.0016 (17)	0.0027 (17)	−0.0013 (17)
O11	0.0463 (14)	0.0556 (17)	0.0504 (17)	−0.0071 (13)	0.0128 (13)	−0.0059 (14)
O12	0.0583 (17)	0.054 (2)	0.0540 (19)	−0.0002 (14)	0.0171 (15)	−0.0058 (14)
Br13	0.0923 (4)	0.0435 (3)	0.0465 (3)	0.0055 (2)	0.0083 (2)	−0.0046 (2)
O14	0.0682 (18)	0.0392 (16)	0.064 (2)	−0.0116 (14)	0.0006 (16)	0.0060 (15)
C15	0.054 (2)	0.040 (2)	0.062 (3)	−0.005 (2)	0.011 (2)	−0.013 (2)
O16	0.071 (2)	0.0481 (19)	0.067 (2)	−0.0012 (16)	−0.0046 (16)	0.0045 (17)
C17	0.067 (3)	0.052 (3)	0.101 (4)	−0.014 (2)	0.015 (3)	−0.014 (3)
Br18	0.0579 (3)	0.0773 (4)	0.0723 (4)	−0.0162 (2)	0.0200 (2)	0.0048 (3)
C19	0.048 (2)	0.052 (3)	0.038 (2)	0.0083 (19)	0.0036 (18)	0.0017 (19)
C20	0.056 (2)	0.075 (3)	0.050 (3)	−0.003 (2)	0.012 (2)	−0.003 (2)
C21	0.064 (3)	0.105 (4)	0.058 (3)	0.003 (3)	0.025 (2)	−0.011 (3)
C22	0.083 (3)	0.094 (4)	0.051 (3)	0.011 (3)	0.024 (3)	−0.020 (3)
C23	0.091 (4)	0.081 (4)	0.065 (3)	−0.015 (3)	0.026 (3)	−0.033 (3)
C24	0.070 (3)	0.066 (3)	0.063 (3)	−0.006 (2)	0.024 (2)	−0.012 (3)

### Geometric parameters (Å, °)

**Table:**

O1—C9	1.365 (4)	O12—H12	0.8200
O1—C2	1.366 (4)	O14—C15	1.383 (5)
C2—C3	1.340 (5)	C15—O16	1.179 (5)
C2—C19	1.461 (5)	C15—C17	1.478 (6)
C3—C4	1.425 (5)	C17—H17A	0.9600
C3—H3	0.9300	C17—H17B	0.9600
C4—O11	1.249 (4)	C17—H17C	0.9600
C4—C10	1.454 (5)	C19—C20	1.385 (5)
C5—O12	1.337 (4)	C19—C24	1.385 (6)
C5—C6	1.388 (5)	C20—C21	1.383 (6)
C5—C10	1.401 (5)	C20—H20	0.9300
C6—C7	1.392 (6)	C21—C22	1.359 (7)
C6—Br13	1.877 (4)	C21—H21	0.9300
C7—C8	1.376 (6)	C22—C23	1.362 (7)
C7—O14	1.387 (4)	C22—H22	0.9300
C8—C9	1.387 (5)	C23—C24	1.376 (6)
C8—Br18	1.878 (4)	C23—H23	0.9300
C9—C10	1.391 (5)	C24—H24	0.9300
			
C9—O1—C2	120.6 (3)	C15—O14—C7	117.5 (3)
C3—C2—O1	120.7 (3)	O16—C15—O14	121.5 (4)
C3—C2—C19	127.2 (4)	O16—C15—C17	128.7 (4)
O1—C2—C19	112.1 (3)	O14—C15—C17	109.8 (4)
C2—C3—C4	122.6 (4)	C15—C17—H17A	109.5
C2—C3—H3	118.7	C15—C17—H17B	109.5
C4—C3—H3	118.7	H17A—C17—H17B	109.5
O11—C4—C3	123.1 (3)	C15—C17—H17C	109.5
O11—C4—C10	121.1 (3)	H17A—C17—H17C	109.5
C3—C4—C10	115.8 (3)	H17B—C17—H17C	109.5
O12—C5—C6	119.5 (4)	C20—C19—C24	118.9 (4)
O12—C5—C10	120.8 (3)	C20—C19—C2	120.5 (4)
C6—C5—C10	119.6 (3)	C24—C19—C2	120.6 (4)
C5—C6—C7	119.6 (4)	C21—C20—C19	119.5 (4)
C5—C6—Br13	120.0 (3)	C21—C20—H20	120.3
C7—C6—Br13	120.4 (3)	C19—C20—H20	120.3
C8—C7—O14	119.2 (4)	C22—C21—C20	121.0 (5)
C8—C7—C6	121.7 (4)	C22—C21—H21	119.5
O14—C7—C6	119.0 (4)	C20—C21—H21	119.5
C7—C8—C9	118.2 (4)	C21—C22—C23	120.0 (5)
C7—C8—Br18	121.3 (3)	C21—C22—H22	120.0
C9—C8—Br18	120.5 (3)	C23—C22—H22	120.0
O1—C9—C8	117.0 (3)	C22—C23—C24	120.3 (5)
O1—C9—C10	121.3 (3)	C22—C23—H23	119.9
C8—C9—C10	121.7 (4)	C24—C23—H23	119.9
C9—C10—C5	119.1 (3)	C23—C24—C19	120.4 (4)
C9—C10—C4	119.0 (3)	C23—C24—H24	119.8
C5—C10—C4	121.9 (3)	C19—C24—H24	119.8
C5—O12—H12	109.5		
			
C9—O1—C2—C3	−2.9 (5)	O1—C9—C10—C4	0.8 (5)
C9—O1—C2—C19	176.1 (3)	C8—C9—C10—C4	−179.1 (3)
O1—C2—C3—C4	2.4 (6)	O12—C5—C10—C9	179.8 (3)
C19—C2—C3—C4	−176.5 (4)	C6—C5—C10—C9	0.7 (5)
C2—C3—C4—O11	178.4 (4)	O12—C5—C10—C4	0.4 (5)
C2—C3—C4—C10	−0.3 (5)	C6—C5—C10—C4	−178.7 (3)
O12—C5—C6—C7	179.8 (3)	O11—C4—C10—C9	180.0 (3)
C10—C5—C6—C7	−1.1 (5)	C3—C4—C10—C9	−1.3 (5)
O12—C5—C6—Br13	−2.3 (5)	O11—C4—C10—C5	−0.6 (6)
C10—C5—C6—Br13	176.8 (3)	C3—C4—C10—C5	178.2 (3)
C5—C6—C7—C8	−0.7 (6)	C8—C7—O14—C15	−95.3 (4)
Br13—C6—C7—C8	−178.6 (3)	C6—C7—O14—C15	89.3 (4)
C5—C6—C7—O14	174.6 (3)	C7—O14—C15—O16	3.5 (6)
Br13—C6—C7—O14	−3.2 (5)	C7—O14—C15—C17	−177.8 (4)
O14—C7—C8—C9	−172.6 (3)	C3—C2—C19—C20	178.7 (4)
C6—C7—C8—C9	2.7 (6)	O1—C2—C19—C20	−0.3 (5)
O14—C7—C8—Br18	7.0 (5)	C3—C2—C19—C24	−0.4 (6)
C6—C7—C8—Br18	−177.7 (3)	O1—C2—C19—C24	−179.4 (4)
C2—O1—C9—C8	−178.8 (3)	C24—C19—C20—C21	−1.1 (6)
C2—O1—C9—C10	1.3 (5)	C2—C19—C20—C21	179.7 (4)
C7—C8—C9—O1	177.0 (3)	C19—C20—C21—C22	0.2 (7)
Br18—C8—C9—O1	−2.6 (5)	C20—C21—C22—C23	0.5 (8)
C7—C8—C9—C10	−3.1 (6)	C21—C22—C23—C24	−0.3 (8)
Br18—C8—C9—C10	177.4 (3)	C22—C23—C24—C19	−0.6 (8)
O1—C9—C10—C5	−178.7 (3)	C20—C19—C24—C23	1.4 (7)
C8—C9—C10—C5	1.4 (5)	C2—C19—C24—C23	−179.5 (4)

### Hydrogen-bond geometry (Å, °)

**Table:**

*D*—H···*A*	*D*—H	H···*A*	*D*···*A*	*D*—H···*A*
O12—H12···O11	0.82	1.86	2.584 (4)	147
C20—H20···O1	0.93	2.34	2.679 (5)	101
C3—H3···O11^i^	0.93	2.57	3.497 (4)	171
C24—H24···O11^i^	0.93	2.48	3.387 (5)	166
C17—H17A···O16^ii^	0.96	2.58	3.309 (6)	133

Symmetry codes: (i) −*x*+1, −*y*−1, −*z*; (ii) *x*, *y*+1, *z*.
